# The Chitosan/Agarose/NanoHA Bone Scaffold-Induced M2 Macrophage Polarization and Its Effect on Osteogenic Differentiation In Vitro

**DOI:** 10.3390/ijms22031109

**Published:** 2021-01-23

**Authors:** Paulina Kazimierczak, Malgorzata Koziol, Agata Przekora

**Affiliations:** 1 Chair and Department of Biochemistry and Biotechnology, Medical University of Lublin, Chodzki 1, 20-093 Lublin, Poland; agata.przekora@umlub.pl; 2 Chair and Department of Medical Microbiology, Medical University of Lublin, Chodzki 1, 20-093 Lublin, Poland; malgorzata.koziol@umlub.pl

**Keywords:** biomaterial, bone regeneration, mesenchymal stem cells, osteoblasts, osteogenic differentiation, co-culture, cytokines

## Abstract

Chronic immune response to bone implant may lead to delayed healing and its failure. Thus, newly developed biomaterials should be characterized by high biocompatibility. Moreover, it is well known that macrophages play a crucial role in the controlling of biomaterial-induced inflammatory response. Immune cells synthesize also a great amount of signaling molecules that regulate cell differentiation and tissue remodeling. Non-activated macrophages (M0) may be activated (polarized) into two main types of macrophage phenotype: proinflammatory type 1 macrophages (M1) and anti-inflammatory type 2 macrophages (M2). The aim of the present study was to assess the influence of the newly developed chitosan/agarose/nanohydroxyapatite bone scaffold (Polish Patent) on the macrophage polarization and osteogenic differentiation. Obtained results showed that macrophages cultured on the surface of the biomaterial released an elevated level of anti-inflammatory cytokines (interleukin-4, -10, -13, transforming growth factor-beta), which is typical of the M2 phenotype. Moreover, an evaluation of cell morphology confirmed M2 polarization of the macrophages on the surface of the bone scaffold. Importantly, in this study, it was demonstrated that the co-culture of macrophages-seeded biomaterial with bone marrow-derived stem cells (BMDSCs) or human osteoblasts (hFOB 1.19) enhanced their osteogenic ability, confirming the immunomodulatory effect of the macrophages on the osteogenic differentiation process. Thus, it was proved that the developed biomaterial carries a low risk of inflammatory response and induces macrophage polarization into the M2 phenotype with osteopromotive properties, which makes it a promising bone scaffold for regenerative medicine applications.

## 1. Introduction

Biomaterials for tissue engineering applications should possess appropriate structural and mechanical characteristics as well as represent the ability to promote cell adhesion, proliferation, and differentiation, avoiding inflammatory reactions after implantation [1]. The chronic immune response may either hinder healing or induce implant loosening and its failure [1,2]. Within the first minutes upon implantation, the surface of the biomaterial is rapidly covered by blood plasma and host tissue proteins (e.g., immunoglobulins, albumin, fibronectin, fibrinogen, vitronectin), causing the settlement of host cells, such as monocytes and fibroblasts, onto the implant surface by interactions primarily with adsorbed proteins [2,3,4]. Then, a few days later, the implant is coated by a layer of fibrotic tissue consisting of collagen, fibroblasts, macrophages, and foreign body giant cells [2,4]. As a result of prolonged inflammation, the biomaterial becomes encapsulated by a dense fibrotic tissue that separates it from the surrounding environment, leading to the failure of osseointegration and implantation. Thus, it is a host immune response that plays a pivotal role in the success of biomaterial implantation [5].

Macrophages are monocyte-derived myeloid cells that play a critical role in the controlling of biomaterial-induced inflammatory response. Macrophages secrete a great amount of signaling molecules that participate in the initiation of inflammatory response to foreign body and regulate cell migration and differentiation, tissue remodeling, and new blood vessel formation [6,7]. The macrophage lineage is characterized by heterogenecity and plasticity. Non-activated macrophages (M0) may be activated (polarized) into two main types of macrophage phenotype: proinflammatory type 1 macrophages (M1) and anti-inflammatory type 2 macrophages (M2) [2,8]. The macrophage phenotype may be changed depending on the cytokine level in the microenvironment. Importantly, each phenotype exerts a different influence on the bone healing process after biomaterial implantation [5]. M1 macrophages may be identified by the secretion of proinflammatory factors, such as interleukin 1 beta (IL-1β), IL-6, IL-12, IL-23, tumor necrosis factor-alpha (TNF-α) as well as by the production of reactive oxygen species (ROS) and inducible nitric oxide synthase (iNOS) [2,5]. Secreted by M1 cells, proinflammatory factors promote the development of Th1 lymphocytes and induce inflammation [9]. In turn, M2 macrophages produce a high level of anti-inflammatory cytokines (including IL-10, IL-4, IL-13) and secrete angiogenic and pro-healing factors e.g., transforming growth factor-beta (TGF-β), vascular endothelial growth factor (VEGF), and fibroblast growth factor-beta (FGF-β), that support the development of Th2 lymphocytes [2,9,10,11]. M2 macrophages promote tissue remodeling and healing as well as support the migration and osteogenic differentiation of mesenchymal stem cells [12,13]. Moreover, various sub-populations of the M2 phenotype may be distinguished, such as M2a, M2b, and M2c, that express different profile of receptors and cytokines [14]. Under in vivo conditions, macrophages may differentiate into different phenotypes depending on the microenvironment and type of the tissue [6]. Under in vitro conditions, macrophages polarization into the M1 phenotype may be induced by lipopolysaccharide (LPS, known also as endotoxin), interferon-gamma (IFN-γ), or macrophage colony-stimulating factor (M-CSF) [5,7,8]. In turn, macrophage transition toward the M2 phenotype may be stimulated by IL-4, IL-13, IL-10, TGF-β, or glucocorticoids [7,8].

After biomaterial implantation, there are several factors that have a great impact on the bone tissue repair, inter alia macrophage–osteoblast cross-talk, environmental soluble factors, and surface properties of the implant [15]. According to the available literature, macrophages may exert the immunomodulatory effect on osteogenic differentiation, inducing bone formation [2,16]. Importantly, the success of biomaterial implantation highly depends on the macrophage polarization induced by the implant. Thus, the aim of this study was to evaluate the influence of the newly developed biomaterial (Patent PL no. 235822) on the macrophage polarization and osteogenic differentiation. The macroporous biomaterial used in this study was composed of chitosan, agarose, and nanohydroxyapatite (chit/aga/HA). The scaffold was previously demonstrated to be biodegradable, highly biocompatible, osteoconductive, and osteoinductive [17,18]. In this study, chit/aga/HA-induced macrophage transition (M1 or M2 phenotype) was identified in a monoculture. The growth of macrophages on the surface of the chit/aga/HA scaffold followed by analysis of the cytokine secretion profile allowed assessing not only inflammatory response to the developed bone scaffold but also the direction of macrophage polarization. Moreover, the macrophages were grown on the surface of the biomaterial, and their effect on osteogenic differentiation of human bone marrow-derived stem cells (BMDSCs) and human osteoblasts (hFOB 1.19) was determined in the co-culture system in vitro.

## 2. Results and Discussion

### 2.1. Monoculture Experiments—Macrophage Characterization

**Figure 1:**
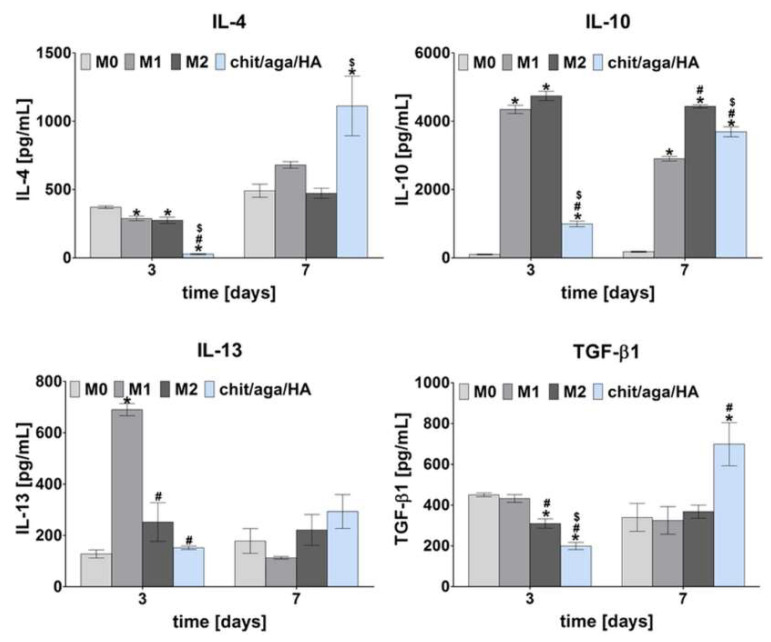
Release profile of anti-inflammatory cytokines (interleukin (IL)-4, IL-10, IL-13, transforming growth factor-beta (TGF-β1)) determined for reference M0, M1, and M2 macrophages as well as macrophages cultured on the surface of chitosan, agarose, and nanohydroxyapatite (chit/aga/HA) biomaterial (*statistically significant results compared to M0 macrophages; ^#^statistically significant results compared to M1 macrophages; ^$^statistically significant results compared to M2 macrophages; *p* < 0.05, one-way ANOVA followed by Tukey’s test).

Biocompatible biomaterials do not induce prolonged inflammation and implant encapsulation by fibrotic tissue [19]. To check inflammatory response to the newly developed bone scaffold—chit/aga/HA, monocyte-derived macrophages were cultured on the surface of the biomaterial for 3 and 7 days and the level of proinflammatory (IL-1β, IL-6, TNF-α), and anti-inflammatory (IL-4, Il-10, IL-13, TGF-β1) factors in the cell culture supernatants were evaluated. M0 macrophages (unpolarized) and macrophages that were polarized in vitro into M1 and M2 phenotype were cultured in the polystyrene wells without the biomaterial. Mentioned cultures served as reference M0, M1, and M2 macrophages to compare the release profile of proinflammatory and anti-inflammatory cytokines with the cytokine release profile of macrophages cultured on the biomaterial. Figure 1 shows the level of anti-inflammatory cytokines produced by all subtypes of the macrophages. Performed analysis showed that after 7 days, macrophages cultured on the surface of the chit/aga/HA released significantly higher amount of IL-4 and TGF-β1 than M0, M1, and M2 macrophages. It is well known that IL-4 belongs to osteotropic factors, playing an important role in the bone metabolism [20]. Moreover, IL-4 down-regulates osteoclast precursors [5]. In turn, TGF-β1 supports osteoblast proliferation and differentiation as well as production of the extracellular matrix (ECM). In addition, a high level of TGF-β1 enhances the expression and secretion of osteoprotegerin (decoy receptor for receptor activator of nuclear factor kappa-Β ligand (RANKL)), suppressing RANKL-RANK-mediated osteoclastogenesis [21]. On the other hand, on the 7th day, macrophages cultured on the chit/aga/HA scaffold released a slightly lower amount of IL-10 compared to M2 macrophages. IL-10 is the anti-inflammatory cytokine that suppresses the synthesis of proinflammatory factors (such as TNF-α, IL-1, and IL-6), collagenase, gelatinase, and nitric oxide. IL-10 may also support osteogenic differentiation [22]. Interestingly, on the 3rd day of culture, M1 macrophages secreted IL-10 at a level similar to M2 macrophages. It is in agreement with studies performed by Tarique et al. [23], who showed that LPS-induced macrophages and INF-γ/LPS-induced macrophages released a greater amount of IL-10 than M0 and M2 macrophages. Moreover, as shown in Figure 1, M1 macrophages significantly elevated the release of IL-13 on the 3rd day of the culture. Nevertheless, on the 7th day, macrophages cultured on the surface of the chit/aga/HA showed higher IL-13 production than M0, M1, and M2 macrophages. IL-13 was found to induce the fusion of macrophages, forming foreign body giant multinucleated cells [7]. Similarly to IL-4, IL-13 suppresses RANKL-induced osteoclastogenesis [24]. Thus, according to the release profile of anti-inflammatory cytokines, it may be assumed that macrophages cultured on the chit/aga/HA biomaterial were predominantly of the M2 phenotype. 

**Figure 2:**
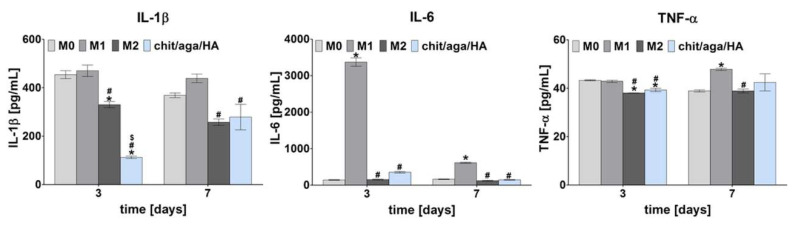
Release profile of proinflammatory cytokines (IL-1β, IL-6, TNF-α) determined for reference M0, M1, and M2 macrophages as well as macrophages cultured on the surface of chit/aga/HA biomaterial (*statistically significant results compared to M0 macrophages; ^#^statistically significant results compared to M1 macrophages; ^$^statistically significant results compared to M2 macrophages; *p* < 0.05, one-way ANOVA followed by Tukey’s test).

Figure 2 presents the release profile of proinflammatory cytokines (IL-1β, IL-6, TNF-α) estimated for M0, M1, and M2 macrophages as well as macrophages cultured on the surface of chit/aga/HA after 3- and 7-day culture. The proinflammatory factors were produced at much higher levels by M1 macrophages compared to other subtypes, which is consistent with the reports available in the literature [8,14,23]. It was observed that macrophages cultured on the biomaterial exhibited a comparable proinflammatory cytokine profile to the reference M2 macrophages, confirming that macrophages cultured on the chit/aga/HA were predominantly of the M2 phenotype. Importantly, macrophages cultured on the chit/aga/HA released a low level of IL-1β and IL-6 (on the 3rd day, the level of IL-1β was even lower than for M2). It is worth noting that a low expression of IL-1β and IL-6 is associated with hindered osteoclast-mediated bone resorption and inhibited acute phase of the immune response [9,25]. Interestingly, all macrophage subtypes and macrophages cultured on the bone scaffold secreted comparable amounts of TNF-α. However, M2 macrophages and macrophages cultured on the biomaterial still released slightly (but with statistical significance) lower amounts compared to M1 macrophages. Lower TNF-α production by macrophages, which were in direct contact with the implant, is an important issue due to its fundamental role in the inflammation and in promoting RANKL-mediated osteoclastogenesis [9,25]. However, it is worth noting that TNF-α was also proved to play an important role in the promotion of angiogenesis, positively affecting bone formation [25].

Analysis of cytokines and growth factors secretion profile showed that M0 macrophages (nonpolarized macrophages) produced simultaneously anti- and proinflammatory factors, indicating a mixed population of M1 and M2 macrophages in the culture (Figure 1 and Figure 2). Macrophages are characterized by flexibility and plasticity, and their phenotype depends on various stimuli from the environment [8]. M1 macrophages are referred to as “classically activated” macrophages, whereas M2 macrophages are referred to as “alternatively activated” macrophages [10,26]. Obtained results clearly proved that chit/aga/HA biomaterial induced M2 polarization of the macrophages, which is desired for accelerated bone regeneration.

**Figure 3:**
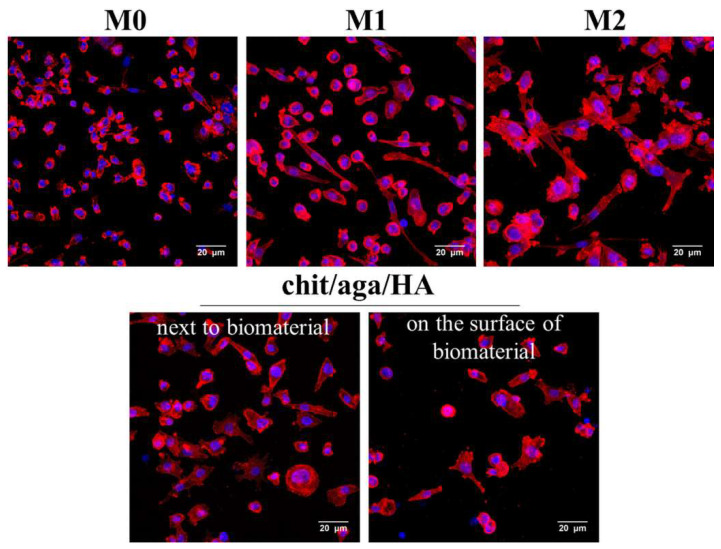
Assessment of macrophage morphology by fluorescent staining of the cell cytoskeleton after 3-day culture in the polystyrene wells (reference M0, M1, and M2 macrophages), on the chit/aga/HA scaffold, and next to the biomaterial (red fluorescence—F-actin filaments, blue fluorescence—nuclei, magnified 400×, scale bar = 20 µm).

M2 macrophage polarization on the surface of the bone scaffold was also confirmed by comparison of cell morphology between various macrophage subtypes. To evaluate the morphology, M0, M1, and M2 macrophages and cells cultured on the surface of the chit/aga/HA scaffold (and also on polystyrene well next to the biomaterial) were stained using fluorescent dyes (fluorescently labeled phalloidin—F-actin filaments, DAPI—nuclei). Stained macrophages were subsequently visualized by confocal laser scanning microscope (CLSM), as shown in Figure 3. Obtained CLSM images showed differences in the morphology (including size and shape of the cells) of respective subtypes of macrophages. Nonpolarized macrophages were mainly small rounded cells. In turn, M1 macrophages appeared as a mix of rounded cells and elongated spindle-shaped cells. Significant differences were observed between M2 and other macrophage subtypes. Namely, M2 macrophages were significantly larger than M0 and M1 cells and were characterized by irregular shape with ragged edges and many filopodia. Importantly, the cytoskeletal staining of macrophages cultured on the chit/aga/HA scaffold and next to the biomaterial showed that their morphology was very similar to the morphology of M2 macrophages. The cells were relatively large and characterized by irregular shape with many filopodia.

In this study, M0 macrophages were polarized into the M1 phenotype upon exposure to LPS/IFN-γ, whereas treatment of the M0 cells with IL-4/IL-13 resulted in the M2 phenotype. Comparative analysis of the cytokine profile and macrophage morphology between references M0, M1, and M2 macrophages and cells grown on the scaffold showed that monocyte-derived macrophages cultured on the surface of the chit/aga/HA exhibited the M2 phenotype. Since the M2 phenotype is characterized by an anti-inflammatory nature, pro-healing activity, and osteopromotive and angiogenic properties, it may be assumed that the chit/aga/HA biomaterial is a promising bone scaffold for regenerative medicine applications, having potential ability to accelerate the bone formation process.

It is worth mentioning that in our previous study [18], it was demonstrated that the chit/aga/HA scaffold has the ability to adsorb large amounts of fibrinogen, which is known to induce M2 macrophage polarization [2]. In this study, the scaffold was presoaked in human blood plasma to simulate conditions occurring after implantation (protein adsorption). Thus, it may be assumed that M2 macrophage polarization was induced by fibrinogen binding to the surface of the chit/aga/HA biomaterial.

### 2.2. Evaluation of Osteogenic Differentiation in Co-Culture System

**Figure 4:**
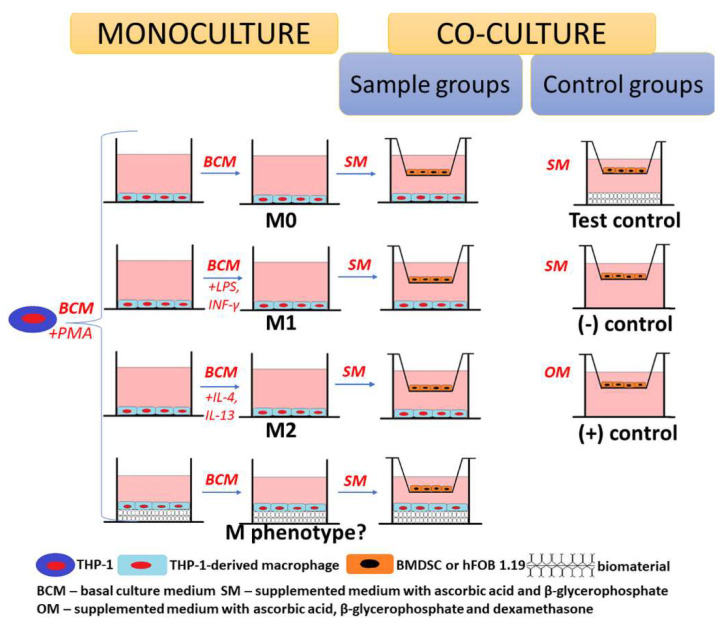
Scheme presenting the main concept of the co-culture experiment.

The monoculture of macrophages on the surface of the chit/aga/HA scaffold demonstrated that the biomaterial induced M2 polarization, which is known to support bone formation. To determine the effect of M2 macrophages on osteogenic differentiation, experiments in a co-culture system were performed. Macrophages grown in the polystyrene wells (reference M0, M1, and M2 macrophages) and on the surface of the chit/aga/HA were co-cultured with human bone marrow-derived mesenchymal stem cells (BMDSCs) or with normal human fetal osteoblasts (hFOB 1.19) (two different co-culture systems were applied) to confirm the immunomodulatory paracrine effect of macrophage phenotypes on osteogenic differentiation and thus bone formation (Figure 4). Importantly, a co-culture system may partially mimic in vivo conditions. It is worth noting that in our previous study, it was found that the chit/aga/HA has osteoinductive properties [18]. Osteoinductivity reflects the ability of the bone scaffold to induce the osteogenic differentiation of mesenchymal stem cells into osteoblasts [2,20]. The osteogenic differentiation process is characterized by three main stages that are associated with the production of specific markers: (I) proliferation phase (Runt-related transcriptional factor 2, type I collagen (Col I), low bone alkaline phosphatase (bALP) activity), (II) extracellular matrix (ECM) synthesis (Col I, Runt-related transcriptional factor 2, Osterix, high bALP activity), and (III) ECM mineralization (osteopontin, osteocalcin (OC), bone sialoprotein, moderate bALP activity) [2,20]. Interestingly, inflammation-associated cells may also exert an osteoinductive impact on mesenchymal stem cells via paracrine signaling and the secretion of cytokines/growth factors [20].

**Figure 5:**
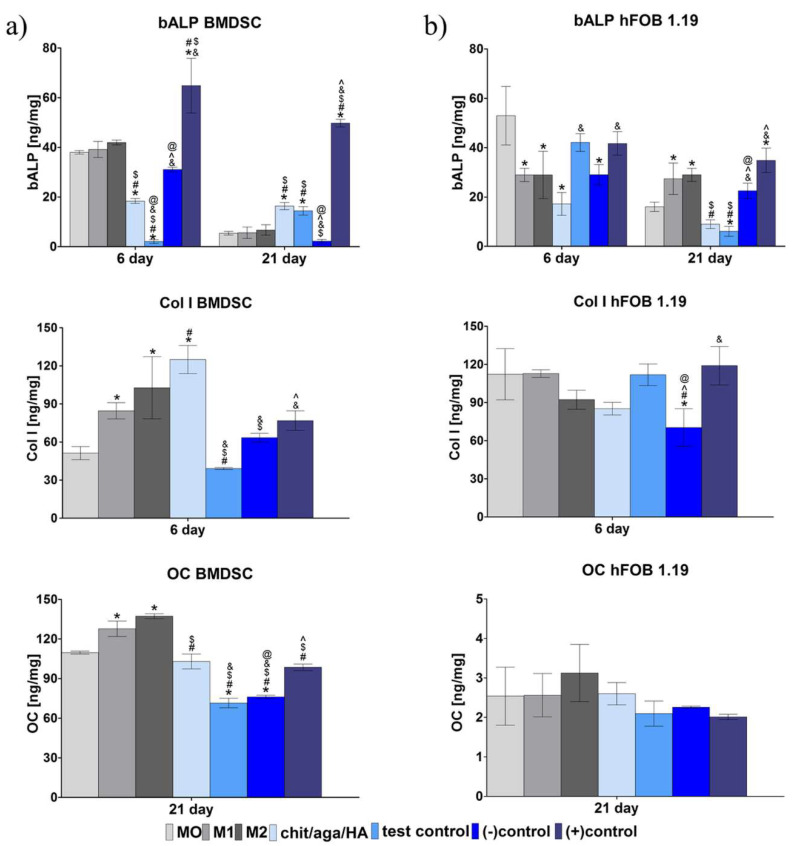
The level of osteogenic markers in (**a**) human bone marrow-derived stem cells (BMDSCs) and (**b**) normal human fetal osteoblasts (hFOB 1.19) co-cultured with M0, M1, and M2 macrophages as well as macrophages grown on the surface of chit/aga/HA biomaterial. The following controls were applied in the experiment: test control—monoculture of BMDSC or hFOB 1.19 cells grown in the presence of chit/aga/HA (without macrophages), (-)control—monoculture of BMDSC or hFOB 1.19 cells in the absence of chit/aga/HA in medium without dexamethasone, (+)control—monoculture of BMDSC or hFOB 1.19 cells in the absence of chit/aga/HA in complete osteogenic medium with dexamethasone. The obtained results were represented as ng of osteogenic marker per mg of total cellular proteins (^*^statistically significant results compared to M0 macrophages; ^#^statistically significant results compared to M1 macrophages; ^$^statistically significant results compared to M2 macrophages; ^&^statistically significant results compared to chit/aga/HA; ^^^statistically significant results compared to test control; ^@^statistically significant results compared to (+) control; ND – not determined because the result was below detection range; *p* < 0.05, one-way ANOVA followed by Tukey’s test).

**Figure 6:**
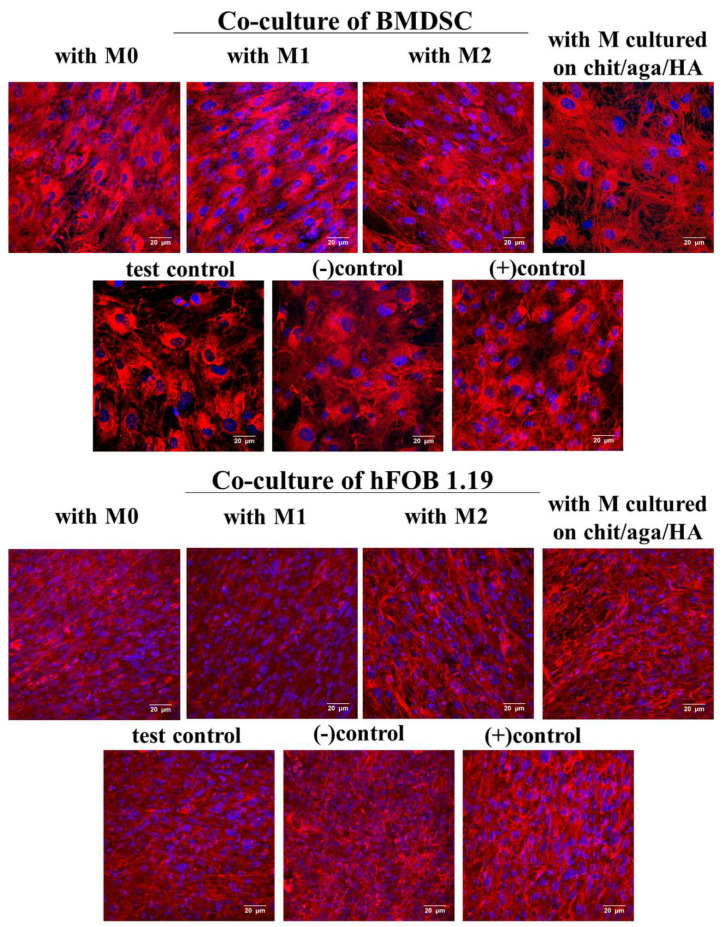
Confocal laser scanning microscope images presenting immunofluorescent staining of type I collagen in the extracellular matrix (ECM) of human bone marrow-derived stem cells (BMDSC) and normal human fetal osteoblast cell line (hFOB 1.19) co-cultured with M0, M1, and M2 macrophages as well as macrophages grown on the surface of chit/aga/HA biomaterial; The following controls were applied in the experiment: test control—monoculture of BMDSC or hFOB 1.19 cells grown in the presence of chit/aga/HA (without macrophages), (-)control—monoculture of BMDSC or hFOB 1.19 cells in the absence of chit/aga/HA in medium without dexamethasone, (+)control—monoculture of BMDSC or hFOB 1.19 cells in the absence of chit/aga/HA in complete osteogenic medium with dexamethasone; red fluorescence—type I collagen, blue fluorescence—nuclei; magnified 400×, scale bar = 20 µm.

Evaluation of the level of typical osteogenic markers in BMDSC and hFOB 1.19 cultures was performed on the 6th (bALP and Col I) and on the 21st (bALP and OC) day of the experiment using appropriate enzyme-linked immunosorbent assays (ELISAs) (Figure 5) and immunofluorescent staining of Col I (Figure 6). Conducted experiments revealed that BMDSCs co-cultured with macrophages-seeded biomaterial (chit/aga/HA sample) produced a significantly higher amount of bALP on the 6th day compared to the monoculture of BMDSCs grown in the presence of chit/aga/HA (sample marked as test control) (Figure 5a). It is worth noting that on the 21st day of the experiment, BMDSCs cultured in the presence of chit/aga/HA—both in monoculture (test control) and co-culture with macrophages (chit/aga/HA sample)—exhibited a significantly higher synthesis of bALP compared to negative (-)control (monoculture of BMDSCs in medium without dexamethasone), confirming the osteoinductive properties of the bone scaffold. Nevertheless, there were no differences in the effect of macrophage subtypes (M0, M1, M2) on the synthesis of bALP in BMDSCs. However, it was observed that M2 macrophages promoted the production of Col I and OC in the culture of BMDSCs. It is well known that M2 macrophages have the ability to release IL-4, which positively affects OC synthesis. This phenomenon was also confirmed by Bastidas-Coral et al. [27], who showed that IL-4 increased OC expression in human adipose tissue-derived stem cells. Interestingly, the level of OC in the chit/aga/HA sample was comparable to positive (+)control (monoculture of BMDSCs in osteogenic medium with dexamethasone) and significantly higher than in the test control and (-)control. In the case of Col I, BMDSCs co-cultured with macrophages-seeded biomaterial (chit/aga/HA sample) produced the highest amounts of this protein among all samples, proving the positive effect of macrophages cultured on the biomaterial on the osteogenic differentiation of stem cells.

Analysis of the level of osteogenic markers in hFOB 1.19 cells co-cultured with macrophages-seeded biomaterial (chit/aga/HA sample in Figure 5b) showed that on the 21st day of the experiment, osteoblasts were in the third stage of osteogenic differentiation (ECM mineralization), since they exhibited moderate bALP activity, reduced levels of Col I, and high levels of OC. In contrast, hFOB 1.19 cultured in the presence of biomaterial without macrophages (sample marked as test control) was still in the second stage of osteogenic differentiation (ECM synthesis) characterized by high Col I production and low or no OC synthesis (Figure 5b). Thus, it may be implied that macrophages cultured on the biomaterial promoted osteogenic differentiation of hFOB 1.19 cells. Importantly, macrophages-seeded chit/aga/HA biomaterial induced the synthesis of OC in hFOB 1.19 cells at a comparable level to M0 cells, M2 macrophages, and (+)control, proving the immunomodulatory effect of macrophages seeded onto the chit/aga/HA scaffold and their M2 phenotype.

CLSM images of Col I showed that BMDSCs and hFOB 1.19 osteoblasts synthesized a great level of Col I protein regardless of the culture conditions (Figure 6). Nevertheless, BMDSCs and hFOB 1.19 cells co-cultured with M2 macrophages and with macrophages-seeded chit/aga/HA biomaterial produced collagen as elongated fibrils that were similar to collagen fibrils produced by (+)controls (BMDSC or hFOB 1.19 cells maintained in osteogenic medium with dexamethasone), confirming the pivotal role of M2 macrophages in the stimulation of osteogenic differentiation, which is consistent with the available reports. According to the literature, macrophages may induce osteogenic differentiation through the BMP-2 signaling pathway [16]. Moreover, Loi et al. [28] revealed that macrophages enhanced the osteogenic differentiation of preosteoblastic MC3T3 cells via enhancing ALP activity, osteocalcin synthesis, and ECM mineralization. Similarly, Wang et al. [29] showed the increased synthesis of osteogenic markers in MC3T3 preosteoblasts in the presence of the immune microenvironment (RAW 264.7 macrophages conditioned medium).

In summary, the co-culture of macrophages-seeded chit/aga/HA biomaterial with BMDSCs or hFOB 1.19 cells increased their osteogenic ability, confirming the immunomodulatory effect of the macrophages on the osteogenic differentiation process. Conducted macrophage polarization in vitro with the use of LPS/IFN-γ and IL-4/IL-13 treatment toward the proinflammatory M1 phenotype and anti-inflammatory M2 phenotype, respectively, allowed evaluating how individual phenotypes modulate the synthesis of typical osteogenic markers. The obtained results showed that the developed chit/aga/HA biomaterial did not increase proinflammatory cytokine production (IL-1β and IL-6, Figure 2) by macrophages but did stimulate anti-inflammatory cytokine release (IL-4, Il-10, IL-13, TGF-β1, Figure 1), which is typical of the M2 phenotype. Moreover, an analysis of cell morphology confirmed the M2 phenotype of the macrophages on the surface of the chit/aga/HA biomaterial (Figure 3). Importantly, in this study, it was demonstrated that the chit/aga/HA scaffold induced an M2 polarization of macrophages that had a positive effect on the osteogenic differentiation of mesenchymal stem cells and osteoblasts (Figure 5 and Figure 6). Therefore, it was confirmed that M2 macrophages play an important role in the bone tissue repair and remodeling [30]. By studying the inflammatory response to the developed bone scaffold, it was also proved that chit/aga/HA carries a low risk of biomaterial-induced inflammation and thus is a very promising scaffold for bone tissue engineering and regenerative medicine applications. Nevertheless, in vivo studies need to be carried out to reliably evaluate the biocompatibility of the chit/aga/HA biomaterial.

## 3. Materials and Methods

### 3.1. Preparation of Biomaterial

The tested chitosan/agarose/nanohydroxyapatite biomaterial (marked as chit/aga/HA) was composed of 2% *w/v* chitosan (50–190 kDa molecular weight, 75–85% deacetylation degree, Sigma-Aldrich Chemicals, Warsaw, Poland), 5% *w/v* agarose (gel point 36 ± 1.5 °C, low EEO, Sigma-Aldrich Chemicals, Warsaw, Poland) and 40% *w/v* nanohydroxyapatite (Sigma-Aldrich Chemicals, Warsaw, Poland), and it was prepared in accordance with the method described previously [17]. Briefly, the suspension of chitosan and agarose prepared in 2% *v/v* acetic acid solution (Avantor Performance Materials, Gliwice, Poland) was mixed with nanohydroxyapatite and sodium bicarbonate (Sigma-Aldrich Chemicals, Warsaw, Poland). The obtained paste was subjected to heating in a water bath (95 °C) and then cooled, frozen, and lyophilized (LYO GT2-Basic, SRK Systemtechnik GmbH, Riedstadt, Germany). The resultant biomaterial was neutralized in 1% *w/v* sodium hydroxide solution (Avantor Performance Materials, Gliwice, Poland), rinsed with deionized water, and air-dried. Prior to the cell culture experiments, the biomaterial was sterilized using ethylene oxide. Subsequently, cylinder-shaped biomaterial discs (2 mm in thick and 10 mm in diameter) were placed in a 24-multiwell plate and preincubated in human blood plasma (obtained from Regional Blood Bank in Lublin, Poland; a volunteer agreed that part of the blood will be used in scientific experiment—written informed consent was obtained) for 3 h in order to imitate a natural environment during the bone regeneration process after implantation of the bone scaffold.

### 3.2. Monoculture Experiments

#### 3.2.1. Macrophage Polarization

Macrophages were obtained by the differentiation of human acute monocytic leukaemia cells (THP-1) in response to phorbol 12-myristate 13-acetate (PMA, Sigma-Aldrich Chemicals, Warsaw, Poland) stimulation. THP-1 cells were obtained from American Type Culture Collection (ATCC-LGC standards, Teddington, UK). The THP-1 cell line is often used as an in vitro model of monocytes and monocyte-derived macrophages due to its similarity to primary monocytes and macrophages [31]. THP-1 cells were cultured in basal culture medium—RPMI-1640 (ATCC-LGC standards, Teddington, UK) containing 0.05 mM 2-mercaptoethanol, 10% fetal bovine serum (FBS), penicillin/streptomycin (100 U/mL and 100 μg/mL, respectively) (Sigma-Aldrich Chemicals, Warsaw, Poland), and maintained at 37 °C in 5% CO_2_ in air atmosphere. The THP-1 cells were seeded into a 24-multiwell plate and onto the biomaterial in 500 μL of a basal culture medium supplemented with 200 nM PMA at a concentration of 1 × 10^6^ cells per well. The addition of PMA to the culture medium induced monocyte differentiation into adherent macrophages [32,33]. After 1 day of culture, adherent THP-1-derived macrophages (non-activated macrophages marked as the M0 phenotype) were polarized to M1 and M2 phenotypes by 3-day exposure to basal culture medium with 100 ng/mL of LPS and 20 ng/mL of INF-γ (Sigma-Aldrich Chemicals, Warsaw, Poland) and basal culture medium with 40 ng/mL IL-4 and 20 ng/mL IL-13 (Sigma-Aldrich Chemicals, Warsaw, Poland), respectively, as shown in Table 1. Macrophages seeded onto the chit/aga/HA biomaterial were cultured in the basal culture medium to assess the influence of the biomaterial on macrophage polarization. Then, all culture media were replaced with a fresh basal culture medium without any factors, and macrophages were cultured for a further 7 days. Every second day, half of the basal culture medium was replaced with a fresh portion.

#### 3.2.2. Macrophage Characterization

The levels of proinflammatory (IL-1β, IL-6, TNF-α) and anti-inflammatory (IL-4, Il-10, IL-13, TGF-β1) cytokines after macrophage polarization were determined after 3 and 7 days of macrophage culture (macrophages were cultured on the chit/aga/HA, whereas reference M0, M1, and M2 cells were cultured in the polystyrene wells). IL-1β, IL-4, IL-6, IL-10, IL-13, TNF-α, and TGF-β1 levels were assessed in the cell culture supernatants using commercially available human-specific ELISAs (EIAab ELISA kit, Wuhan, China). ELISAs were conducted according to the manufacturer protocol. Additionally, after 7 days of macrophages culture, evaluation of cell morphology was performed. The macrophages were fixed with 3.7% paraformaldehyde, permeabilized with 0.2% Triton X-100, and blocked with 1% bovine serum albumin (Sigma-Aldrich Chemicals, Warsaw, Poland), and then incubated for 30 min at room temperature with staining solution containing DAPI (Sigma-Aldrich Chemicals, Warsaw, Poland) and AlexaFluor635-conjugated phallotoxin (Invitrogen, Carlsbad, California, CA, USA). The DAPI (blue fluorescence) and phalloidin (red fluorescence) stain nuclei and cytoskeletal filaments (F-actin), respectively. Stained macrophages were visualized by confocal laser scanning microscope (CLSM, Olympus Fluoview equipped with FV1000, Olympus Polska Sp. z o. o., Warsaw, Poland).

### 3.3. Co-Culture Experiments

#### 3.3.1. Cells

The co-culture experiments were conducted using THP-1-derived macrophages, normal human fetal osteoblast cell line (hFOB 1.19, ATCC-LGC standards, Teddington, UK), and human bone marrow-derived stem cells (BMDSCs, ATCC-LGC standards, Teddington, UK). The THP-1 cells were cultured as described in Section 3.2.1. The hFOB 1.19 cells were maintained in a 1:1 mixture of DMEM/Ham’s F12 medium without phenol red (Sigma-Aldrich Chemicals, Warsaw, Poland) containing 10% FBS, penicillin/streptomycin (100 U/mL and 100 μg/mL, respectively), and incubated at 34 °C in 5% CO_2_ in air atmosphere. The BMDSCs were maintained in Mesenchymal Stem Cell Basal Medium (ATCC-LGC Standards, Teddington, UK) containing a Bone Marrow-Mesenchymal Stem Cell Growth Kit (ATCC-LGC Standards, Teddington, UK), penicillin/streptomycin (10 U/mL and 10 μg/mL, respectively) and incubated at 37 °C in 5% CO_2_ in air atmosphere.

#### 3.3.2. Co-Culture System Design

THP-1-derived macrophages growing in polystyrene wells (M0, M1, and M2 macrophages) and onto the surface of the chit/aga/HA were co-cultured with BMDSC or with hFOB 1.19 cells to confirm the paracrine effect of macrophages on osteogenic differentiation. The differentiation of THP-1 monocytes into adherent THP-1-derived macrophages and their polarization to the M1 and M2 phenotypes was conducted in the same way as described in Section 3.2.1. Briefly, the THP-1 cells were seeded at a concentration of 1 × 10^6^ cells per well into a 24-multiwell plate and onto the biomaterial in 500 μL of a basal culture medium (RPMI-1640 supplemented with 10% FBS, 0.05 mM 2-mercaptoethanol, 100 U/mL penicillin, 100 μg/mL streptomycin) supplemented with 200 nM PMA. After 1 day of culture, adherent THP-1-derived macrophages (M0 phenotype) were polarized to M1 and M2 phenotypes by 3-day exposure to appropriate culture medium (Table 1). Then, the media from the macrophages culture were removed, and cell culture inserts with 1 μm pore size were placed above the macrophage layer. The BMDSCs and hFOB 1.19 cells were seeded into the inserts at a concentration of 1 × 10^5^ cells per sample. The co-cultured cells were maintained in the complete culture medium supplemented with 50 μg/mL ascorbic acid, 10 mM β-glycerophosphate (Sigma-Aldrich Chemicals, Warsaw, Poland), and 0.05 mM 2-mercaptoethanol. Moreover, three different control groups were applied in the experiment: (1) test control—BMDSCs or hFOB 1.19 cells cultured in the inserts without a macrophage layer on the well bottom but with chit/aga/HA biomaterial (assessment of the biomaterial effect on osteogenic differentiation), (2) negative control of osteogenic differentiation (marked as (-)control)—BMDSCs or hFOB 1.19 cells cultured in the inserts without macrophages and biomaterial (assessment of the level of osteogenic markers without induction of bone formation using dexamethasone), (3) positive control of osteogenic differentiation (marked as (+)control)—BMDSCs or hFOB 1.19 cells cultured in the inserts without macrophages and biomaterial and maintained in osteogenic medium additionally supplemented with 10^−7^ M dexamethasone to induce bone formation (Sigma-Aldrich Chemicals, Warsaw, Poland) to induce osteogenic differentiation (Figure 4, Table 2). The co-culture experiment was performed for 21 days, and half of the media was exchanged every 3rd day.

#### 3.3.3. Evaluation of Osteogenic Differentiation in Co-Culture

On the 7th and 21st day of the co-culture system experiment, quantitative evaluation of osteogenic markers in the BMDSC and hFOB 1.19 cell lysates was performed using commercially available human-specific ELISAs: type I collagen (Col I), osteocalcin (OC) (EIAab ELISA kit, Wuhan, China), and bone alkaline phosphatase (bALP) (FineTest ELISA Kit, Wuhan, China). The cell lysates were prepared in accordance with the method described previously [34] via two freeze–thaw cycles and sonification (ultrasonic processor UP100H, Hielscher Ultrasound Technology, Teltow, Germany) for 30 s at 30% amplitude. Additionally, immunofluorescent staining of Col I in ECM of BMDSC and hFOB 1.19 cells was carried out on the 6th day. The immunofluorescent staining was performed in accordance with the procedure described previously [35]. In brief, the cells were incubated overnight at 4 °C with primary human-specific anti-collagen I antibodies (Col1a1/Col1a2, Abnova, Taoyuan City, Taiwan) at a concentration of 10 µg/mL and then incubated for 1 h at room temperature with secondary antibodies Alexa-Fluor^®^647 donkey anti-goat IgG antibody (Abcam, Cambridge, UK) at a concentration of 2 µg/mL. Additionally, the cell nuclei were labeled with the DAPI. Stained cells were observed using CLSM.

## 4. Patents

The method for the production of the chit/aga/HA biomaterial is protected by Polish Patent no. 235822: *Cryogel bone scaffold based on chitosan and bioceramics and the method for its production*

## Figures and Tables

**Table 1:** Culture media that were applied during macrophage polarization.

Type of Macrophages Culture	Applied Culture Medium
M0 phenotype	Basal culture medium (RPMI-1640 supplemented with 10% FBS, 0.05 mM 2-mercaptoethanol, 100 U/mL penicillin, 100 μg/mL streptomycin)
M1 phenotype	Basal culture medium supplemented with 100 ng/mL LPS and 20 ng/mL INF-γ
M2 phenotype	Basal culture medium supplemented with 40 ng/mL IL-4 and 20 ng/mL IL-13
Macrophages cultured on the surface of the chit/aga/HA	Basal culture medium

**Table 2:** Experimental conditions of control groups used in the co-culture experiment.

Type of Control Groups	Experimental Conditions	
	Type of Cells (Monoculture)	Medium
Test control	BMDSCs or hFOB 1.19 cells cultured in the presence of biomaterial	Supplemented medium (complete culture medium supplemented with 50 μg/mL ascorbic acid, 10 mM β-glycerophosphate, and 0.05 mM 2-mercaptoethanol)
Negative control (marked as (-)control)	BMDSCs or hFOB 1.19 cells	Supplemented medium (complete culture medium supplemented with 50 μg/mL ascorbic acid, 10 mM β-glycerophosphate, and 0.05 mM 2-mercaptoethanol)
Positive control (marked as (+)control)	BMDSCs or hFOB 1.19 cells	Osteogenic medium (complete culture medium supplemented with 50 μg/mL ascorbic acid, 10 mM β-glycerophosphate, 0.05 mM 2-mercaptoethanol, and 10^−7^ M dexamethasone)

## Data Availability

The raw/processed data required to reproduce these findings can be obtained from the corresponding author (paulina.kazimierczak@umlub.pl) upon reasonable request.

## Abbreviations

bALP	Bone alkaline phosphatase
chit/aga/HA	Chitosan/agarose/nanohydroxyapatite
CLSM	Confocal laser scanning microscope
Col I	Type I collagen
ECM	Extracellular matrix
ELISAs	Enzyme-linked immunosorbent assays
IL	Interleukin
IFN-**γ**	Interferon gamma
iNOS	Inducible nitric oxide synthase
LPS	Lipopolysaccharide
M-CSF	Macrophage colony-stimulating factor
ROS	Reactive oxygen species
OC	Osteocalcin
TGF-β	Transforming growth factor beta
TNF-α	Tumor necrosis factor alpha
VEGF	Vascular endothelial growth factor
